# Dynamic evolution of schistosomiasis distribution under different control strategies: Results from surveillance covering 1991–2014 in Guichi, China

**DOI:** 10.1371/journal.pntd.0008976

**Published:** 2021-01-06

**Authors:** Yi Hu, Robert Bergquist, Yue Chen, Yongwen Ke, Jianjun Dai, Zonggui He, Zhijie Zhang

**Affiliations:** 1 Department of Epidemiology and Biostatistics, School of Public Health, Fudan University, Shanghai, China; 2 Key Laboratory of Public Health Safety, Ministry of Education, Shanghai, China; 3 Laboratory for Spatial Analysis and Modeling, School of Public Health, Fudan University, Shanghai, China; 4 Ingerod, Brastad, Sweden; 5 School of Epidemiology, Pubic Health and Preventive Medicine, Faculty of Medicine, University of Ottawa, Ottawa, Ontario, Canada; 6 Schistosomiasis Station of Prevention and Control in Guichi Distirct, Anhui Province, China; PUCRS, BRAZIL

## Abstract

**Background:**

Since the founding of the China, the Chinese government, depending on the changing epidemiological situations over time, adopted different strategies to continue the progress towards elimination of schistosomiasis in the country. Although the changing pattern of schistosomiasis distribution in both time and space is well known and has been confirmed by numerous studies, the problem of how these patterns evolve under different control strategies is far from being understood. The purpose of this study is, therefore, to investigate the spatio-temporal change of the distribution of schistosomiasis with special reference to how these patterns evolve under different control strategies.

**Methodology / Principal findings:**

Parasitological data at the village level were obtained through access to repeated cross-sectional surveys carried out during 1991–2014 in Guichi, a rural district along the Yangtze River in Anhui Province, China. A hierarchical dynamic spatio-temporal model was used to evaluate the evolving pattern of schistosomiasis prevalence, which accounted for mechanism of dynamics of the disease. Descriptive analysis indicates that schistosomiasis prevalence displayed fluctuating high-risk foci during implementation of the chemotherapy-based strategy (1991–2005), while it took on a homogenous pattern of decreasing magnitude in the following period when the integrated strategy was implemented (2006–2014). The dynamic model analysis showed that regularly global propagation of the disease was not present after the effect of proximity to river was taken into account but local pattern transition existed. Maps of predicted prevalence shows that relatively high prevalence (>4%) occasionally occurred before 2006 and prevalence presents a homogenous and decreasing trend over the study area afterwards.

**Conclusions:**

Proximity to river is still an important determinant for schistosomiasis infection regardless of different types of implemented prevention and control strategies. Between the transition from the chemotherapy-based strategy to the integrated one, we noticed a decreased prevalence. However, schistosomiasis would remain an endemic challenge in these study areas. Further prevention and control countermeasures are warranted.

## Author summary

Schistosomiasis japonica is one of the most serious parasitic diseases in China. The Chinese government has launched three different rounds of national schistosomiasis control programs since 1950s. The latest two are the World Bank Loan Project (WBLP) that ushered in chemotherapy as the main control approach, active from 1992 to 2001, and the integrated control strategy that took its place in 2005. In this study, we investigated changes in the dynamics of schistosomiasis transmission over space and time under these different control strategies. Based on spatio-temporal analyses of the schistosomiasis prevalence data at the village level during 1991–2014 in Guichi, Anhui Province, we built a dynamic model to evaluate the evolving pattern of prevalence. We found that the schistosomiasis prevalence generally showed a north-western shift over the study area during 1991–2005, while there was no such trend during 2006–2014. This global shifting trend disappeared after the effect of proximity to river was taken into account, but local change still existed which was possibly due to the transition between the two latest national control strategies. We conclude that proximity to River is still an important determinant for schistosomiasis prevalence and that although the integrated control strategy is more effective than the WBPL in reducing schistosomiasis prevalence, the disease would remain endemic for the long term without further improvements of the control program.

## Introduction

*Schistosomiasis japonica*, caused by the trematode worm *Schistosoma japonicum* [1], is responsible for human and animal infections in China, the Philippines and parts of Indonesia [2]. It has a documented history of at least 2100 years along the Yangtze River Basin and once had considerable public health and economic significance in the regions [3]. After the founding of the China, the central government made great strides toward fighting schistosomiasis. The initial national schistosomiasis control program began in 1950s [4]; it mainly based on control of the intermediate snail host that spreads the infection to the definitive mammal host, but chemotherapy was also used though the drugs available at the time were not very effective. Strong political will and sufficient long-term financial support [5] resulted in a remarkable decline in both prevalence and intensity of disease [6,7].

Despite the success of mass treatment using praziquantel trough the 10-year World Bank Loan Project (WBLP) that started in 1992, schistosomiasis rebounded in the early 2000s, not only because of the termination of the WBLP [8,9], but also due to common floodings along the Yangtze River basin [10] and other natural, social and economic factors [11,12]. A new national strategy integrating chemotherapy, snail control, health education, improved sanitation and access to safe water, was initiated in 2005 [13,14]. This change of strategy achieved success almost immediately in controlling *S*. *japonicum* transmission, as it again resulted in lower numbers of infections, both in domestic animals and humans [15,16].

Uneven schistosomiasis distribution is a well-known phenomenon, even in limited geographical settings [17,18]. Numerous studies have identified this focal pattern [19,20,21,22,23], which is representative for the distribution of schistosomiasis cases in both time and space. However, the question how this pattern evolves is far from understood. The role of, and the causes leading to, epidemiological pattern transition that results in spread of diseases have only recently become a major concern for epidemiological studies [24]. Considering the substantial changes the national strategy of schistosomiasis control in China has undergone, we assume that corresponding changes in the local environmental conditions in the endemic areas have taken place, resulting in pattern transitions of the disease. Understanding and quantifying these transitions would help assessing the effectiveness of control strategies [25,26].

The goal of this study was therefore to investigate the evolution of schistosomiasis distributions patterns under the two latest stages of the national program for schistosomiasis control.

## Materials and methods

### Ethics statement

Approval for oral consent and other aspects of the surveys was granted by the Ethics Committee of Fudan University (ID: IRB#2017-09-0635). Written informed consent was also obtained from adult participants (≥18 years). Parental consent was sought for participants aged 5 to 17 years. All the individual information of participants were anonymized by deleting the personal identifiers (such as name, parent name, home address, and telephone number) for the purpose of protecting patients’ privacy. All information and consent procedures were conducted in Guichi, Anhui Province, China.

We first conducted an exploratory data analysis of schistosomiasis prevalence in Guichi, a rural district belonging to the city of Chizhou, Anhui Province, using annual village-level prevalence data for the period 1991–2014, and then built a dynamic model to investigate the evolution of schistosomiasis epidemiology during this period.

### Study area

**Fig 1:**
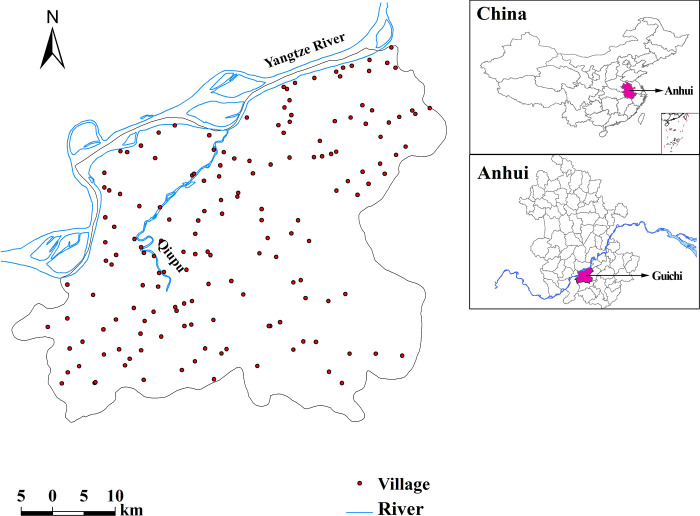
Endemic area of schistosomiasis in Guichi, Anhui Province, China. The Yangtze River is seen along the northern county border, while the Qiupu River runs within the county. This figure was produced in ArcGIS 10.0 (ESRI, Redlands, CA, USA) using shape files representing Guichi, Anhui, and China’s current administrative units (obtained from the geographic data at https://www.naturalearthdata.com/).

Guichi remains one of the most highly endemic areas of schistosomiasis in China and became, for this reason, one of the 20 national sentinel surveillance sites in 2000 [27]. Situated in the middle-lower reaches of the Yangtze River Basin, Guichi spans approximately 2,500 km^2^ and is composed of 164 villages. Besides the Yangtze River, another major river, the Qiupu, runs across the region from the Midwest to the North (Fig 1). The humid subtropical, monsoon climate, with an average annual temperature about 16°C and annual rainfall around 1,600 mm, provide an ideal environment for the survival of *O*. *hupensis*, the intermediate snail host [20].

### Parasitological data

*S*. *japonicum* infection prevalence data for 1991–2014 were provided by the local anti-schistosomiasis station in Guichi. These data were originally collected through village-based field surveys that required residents aged 5 to 65 years to participate in. Indirect Hemagglutination Assay (IHA) test was first used to screen the individuals according to the manufacturer’s instructions and then the parasitological test was applied to the seropositive individuals by reading three Kato-Katz [28] thick smears from one stool specimen. All those found to be egg-positive were regarded as having an active *S*. *japonicum* infection and therefore treated with praziquantel. For each year, we removed the villages for statistically reliable analysis, in which the number of individuals participating in the serological test are less than 100. With this criteria, the number of sample village ranged from 64 in 1995 to 128 in 2011.

### Environmental data

Water body data, including the Yangtze River and Qiupu River, were extracted from the World Wildlife Fund’s Conservation Science Data Sets (http://worldwildlife.org). For each sample village, the Euclidian distance (unit: kilometer) to the nearest river was calculated by using ArcGIS software version 10.0 (ESRI Inc., Redlands, CA, USA).

### Statistical analysis

With these large spatio-temporal schistosomiasis prevalence data, we carried out an exploratory data analysis to give hints to follow-up spatio-temporal dynamic modeling. A Hovmoller plot, a two-dimensional space-time visualization in which space is collapsed onto one dimension and where the second dimension denotes time [29], was employed.

### Dynamic spatio-temporal model

In order to model the dynamic pattern of schistosomiasis, we assume that there is a true unobserved spatio-temporal process hidden behind the yearly observed prevalence in each village, which is incorporated into the framework of a hierarchical model. The basic representation of the hierarchical model is typically composed of data level (whose conditional probability distribution given processes and parameters is independent), process level (which determines change of data level given parameters), and parameter level (which are contained in the previous two levels).

#### Data level

This describes the relationship between the observations and the latent spatio-temporal process. Specifically, we could present the data model as:

$$Z_{t}=\alpha +\sum_{k=1}^{m}\beta_{k}X_{k}+H_{t}Y_{t}+\varepsilon_{t},\;t=1,\ldots ,T$$ (1)

where *Z*_*t*_ corresponds to the data vector (which includes arbitrary spatial locations) at time *t*, α is the intercept, *X*_*kt*_ are fixed covariates that specified as environmental or socio-economic factors and *β*_*kt*_ is the *k*th covariate’s coefficient, *Y*_*t*_ is the latent spatio-temporal process, with a linear mapping here, *H*_*t*_ that connects the data to the latent process, and finally *ε*_*t*_ is a time-varying (but statistically independent in time) continuous mean-zero Gaussian process which is typically measurement error. Although it is in principle possible to parameterize the mapping matrix *H*_*t*_ and/or estimate it directly in some cases, we shall typically assume that it is known as a simple incidence matrix (i.e., a matrix of ones and zeros) which depends on the dimension of *Y*_*t*_. In addition, an important assumption is that the data are independent (in time) when conditioned on the latent process *Y*_*t*_ and parameters *α*_*t*_, *H*_*t*_, and *ε*_*t*_.

#### Process level

The latent process here is presumed to follow a first-order linear Markovian spatio-temporal process which assumes that the value of the process at a given location at the present time is made up of a weighted combination of the process throughout the spatial domain at previous times, plus an additive, Gaussian, spatially coherent “innovation”. This is perhaps best represented in a continuous-spatial context through an integro-difference equation (IDE) as follows:

$$Y_{t}(s)=\int_{D_{s}}m(s,x;\theta_{p})Y_{t-1}(x)dx+\omega_{t}(s),\;s,x\in D_{s},\;t=1,2,\ldots ,$$ (2)

where *m*(*s*,*x*;*θ*_*p*_) is a transition kernel, depending on parameters *θ*_*p*_ that specify "redistribution weights" for the process time over the spatial domain *D*_*s*_, and *ω*_*t*_(∙) is a time-varying (but statistically independent in time) continuous mean-zero Gaussian spatial process independent of *Y*_*t*−1_(∙). Elements {*m*(*s*,*x*;*θ*_*p*_)} consist of the "so called" transition matrix *M*. We assume *m*(*s*,*x*;*θ*_*p*_) to be a Gaussian-shape kernel as a function of *x* relative to the location *s*:

$$m(s,x;\theta_{p})=\theta_{p,1}(s)exp(-\frac{1}{\theta_{p,2}(s)}[{\left(x_{1}-\theta_{p,3}(s)-s_{1}\right)}^{2}+{\left(x_{2}-\theta_{p,4}(s)-s_{2}\right)}^{2}]$$ (3)

where the kernel amplitude is given by *θ*_*p*,1_, the length-scale (variance) parameter *θ*_*p*,2_ corresponds to kernel scale (aperture) parameter, the mean (shift) parameter *θ*_*p*,3_ and *θ*_*p*,4_ correspond to a shift of kernel relative to location *s*, and 1 and 2 (∙ corresponds to *s* and *x*) denotes longitude and latitude coordinate, respectively. The kernel parameters *θ*_*p*_ in formula (3) are spatially varying which would result in complex dynamic behavior, but they can be spatially invariant as well (more details in S1 Text). In addition, we assume here that *θ*_*p*_ dose not vary with time. In general, $\int_{D_{s}}m(s,x;\theta_{p})dx<1$ is needed for the process to be stable (i.e., non-explosive) in time.

#### Parameter level

At this level, we summarize all the parameters to be estimated in above models, which include the intercept *α*, the fixed-effect coefficient for proximity to river *β*, and kernel parameters *θ*_*p*_, and variances of the measurement error *ε*_*t*_ and the latent process error *ω*_*t*_. All the parameters are estimated using the expectation-maximization (EM) algorithm [30] and inference for the latent process *Y*_*t*_ is implemented using kalman filtering and smoothing [31]. Parametric significance (*p*-value) was calculated using a permutation approach with 99 random simulations of the observed prevalence.

**Fig 2:**
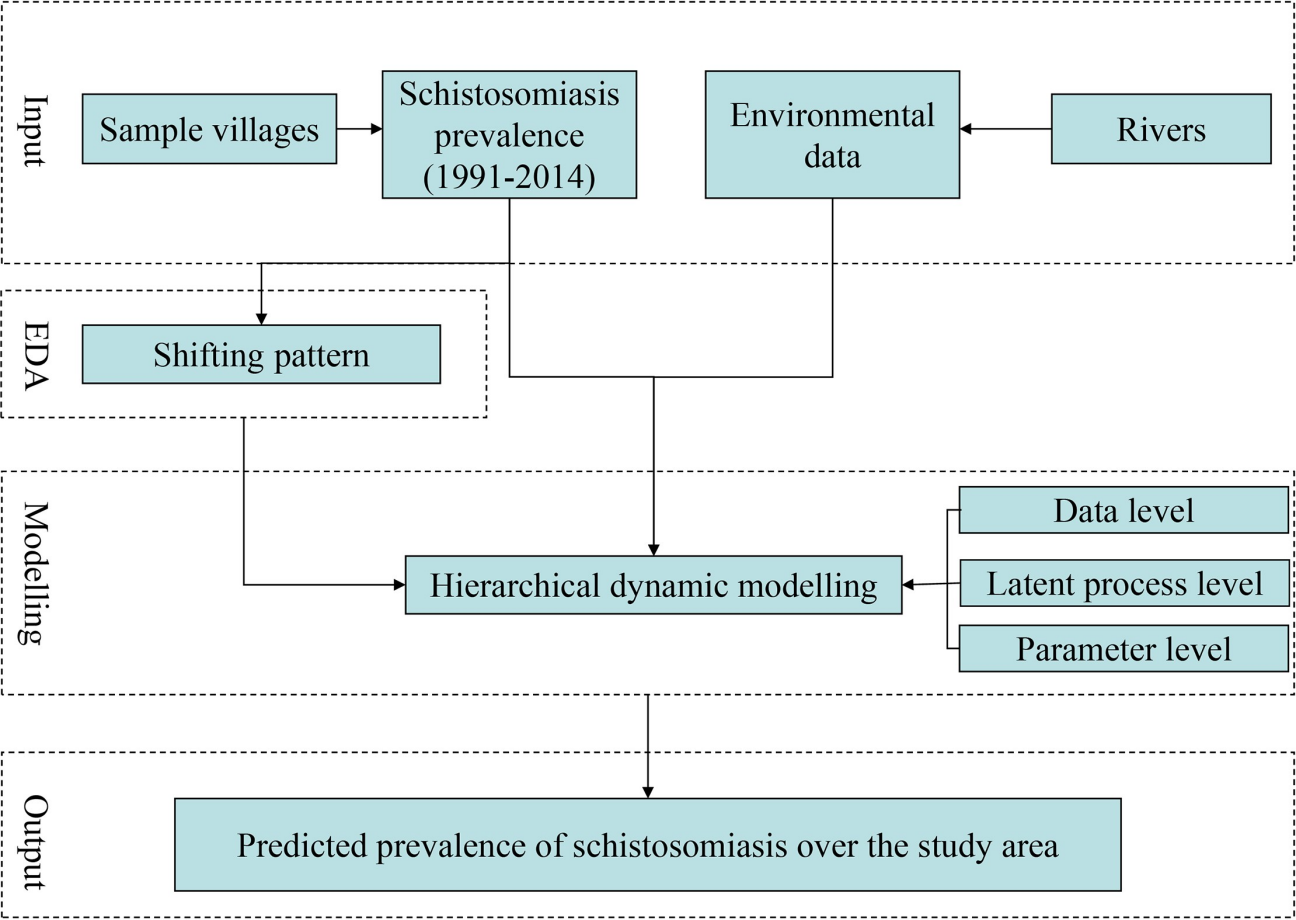
Workflow of data analysis. EDA: exploratory data analysis.

For model validation, we utilize a k-fold cross-validation (k is assumed to be 10 in our study) in terms of prediction performance as follows to determine whether spatially invariant or spatially varying kernel can capture the dynamic behavior of the hidden process *Y*_*t*_:

$$MSPE=\frac{1}{Tm}\sum_{j=1}^{T}\sum_{i}^{m}{\left\{Z_{v}(s_{i};t_{j})-\hat{Z_{v}(s_{i};t_{j})}\right\}}^{2}$$ (4)

where {*Z*_*v*_(*s*_*i*_;*t*_*j*_)} are samples of observations by randomly sampling 10% of all the spatial and temporal observation data and $\left\{\hat{Z_{v}(s_{i};t_{j})}\right\}$ are prediction by the dynamic model based on the rest of the observation. All model fitting was performed using R software, specifically the IDE package (R Development Core Team, 2013). Finally, the latent process, in the form of predicted schistosomiasis prevalence over the study area (displayed as maps), were produced using the optimal model; the spatial resolution for prediction was 1 km. A conceptual framework of the data analysis is shown in Fig 2.

## Results

**Fig 3:**
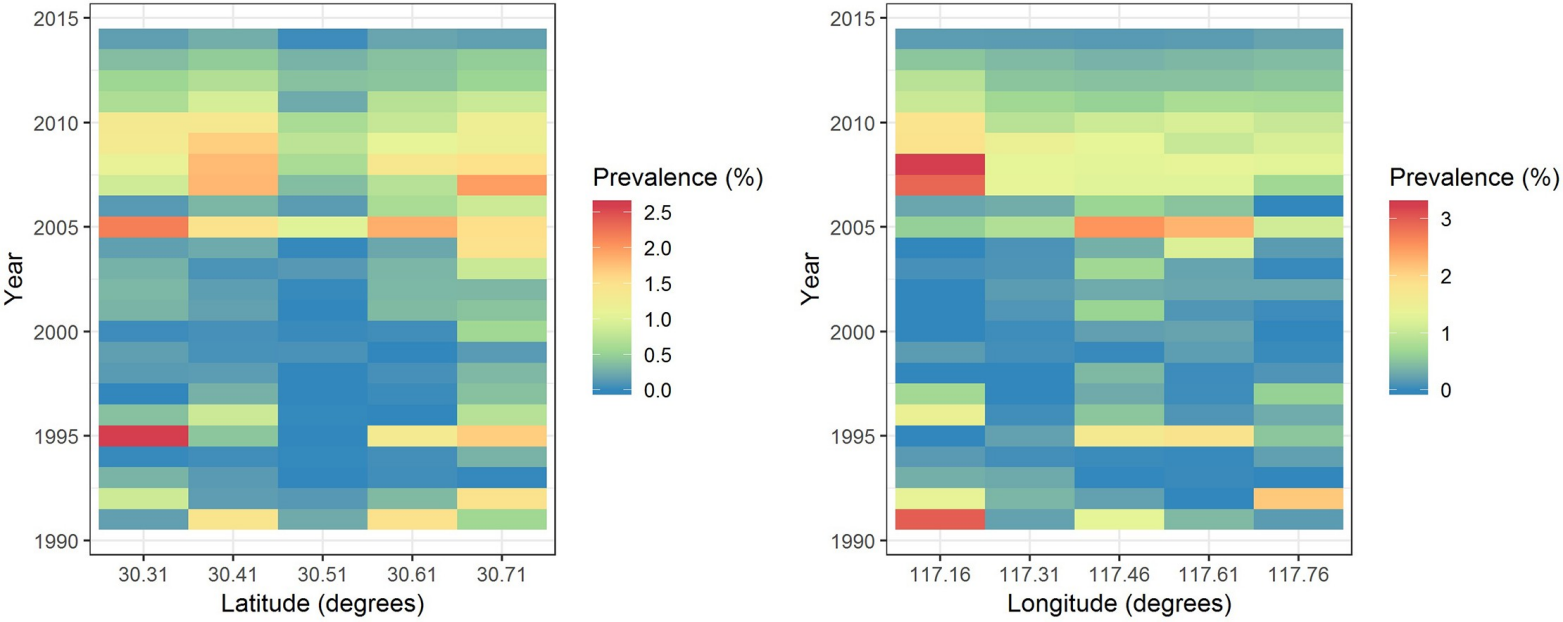
Hovmoller plots for latitude (left) and longitude (right) coordinates with respect to schistosomiasis prevalence in Guichi, Anhui Province, China. The color denotes schistosomiasis prevalence in percentage (%).

Fig 3 shows Hovmoller plots for the schistosomiasis prevalence in the study area between 1991 and 2014. Spatially, we see in the left panel that the prevalence increases generally as we move northwards within the county (corresponding to higher values for the latitudes), particularly as we reach the latitude 30.71 degree, but there is no trend with respect to the longitude (right panel) before 2006. Temporally, we find out a potential temporal trend in prevalence with decreasing longitude in the right panel (e.g., the yellow color at the right bottom in the plot slants at a left-top direction from 1991 to 1996 and the same phenomena from 2007 to 2010). In addition, the prevalence seems to be increasing since 2005 but then decrease again after 2010.

**Fig 4:**
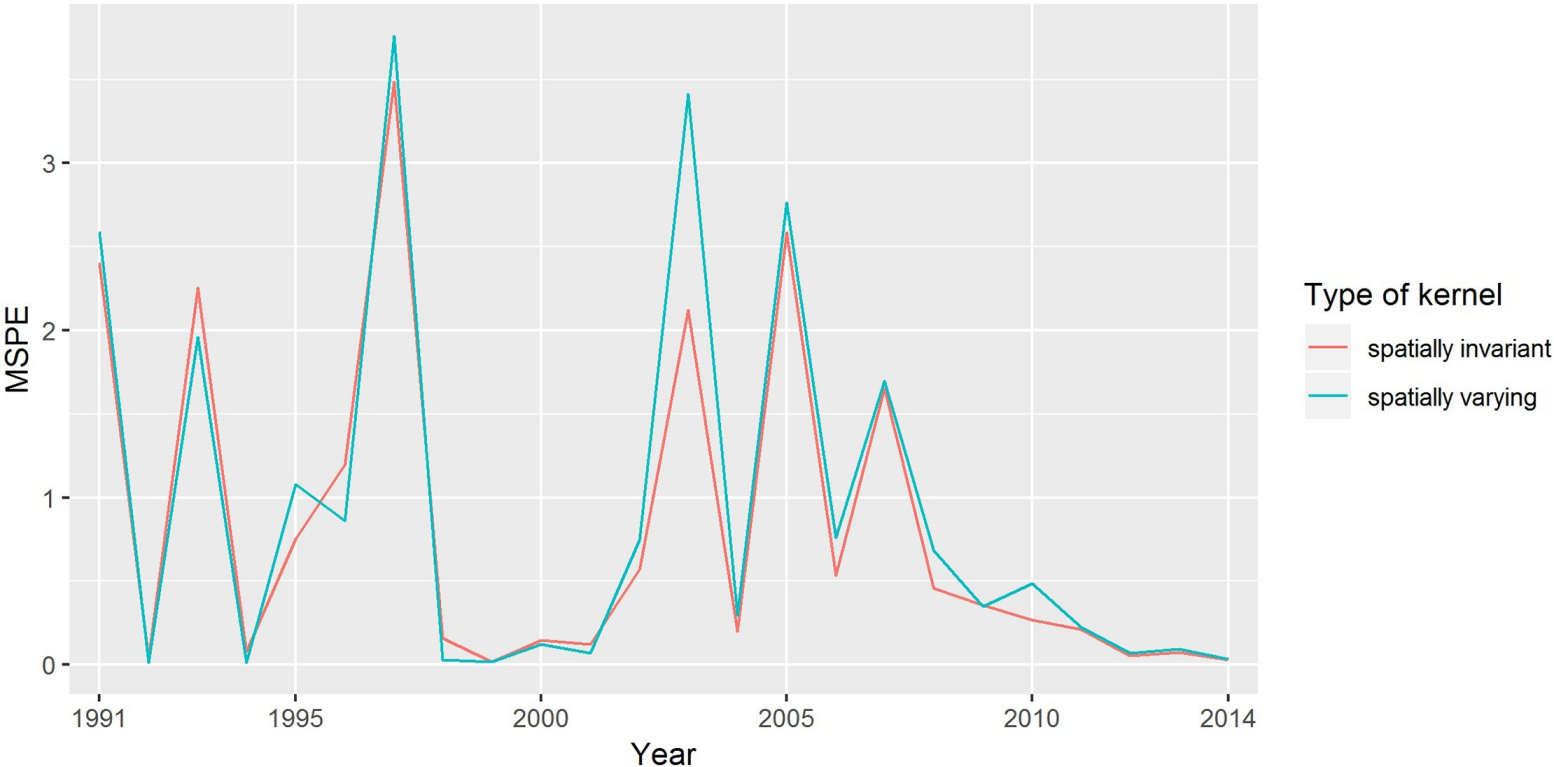
Average MSPE of the schistosomiasis prevalence predictions with the spatially invariant (red) and spatially varying (green) kernel as a function of time.

Fig 4 illustrates the average MSPE of predictions with the spatially invariant and spatially varying kernels at each year, respectively. As shown in this figure, the spatially invariant kernel *θ*_*p*_ fit spatio-temporal prevalence data better (less MSPE at almost each year and the whole study period). The EM estimates for all parameters in spatially invariable model are presented in Table 1. There was a statistically negative correlation (*p*-value = 0.01) between schistosomiasis prevalence and proximity to river. The very small value of shift parameters (*θ*_*p*,3_ = 3.68e(-03) and *θ*_*p*,4_ = 0.38e(-03)) indicates that there was almost no presence of shift in the disease data. Table 2 shows that eigenvalues of the transition matrix *M* of the spatially invariant model are all less than one, indicating stationary patterns.

**Table 1:** The point expectation-maximization (EM) estimates of parameters for the spatially invariant model.

Parameters	Estimate	*p*-value
Intercept	1.525	0.00
Proximity to river	-0.027	0.01
*θ*_*p*,1_	2.483e3	0.34
*θ*_*p*,2_	0.102e(-03)	0.27
*θ*_*p*,3_	3.676e(-03)	0.58	
*θ*_*p*,4_	0.377 e(-03)	0.50	

Variances of the measurement error *ε*_*t*_ and the latent process error *ω*_*t*_ are not available in the current version of IDE package (R Development Core Team, 2013).

**Table 2:** Statistics for eigenvalues of the transition matrix *M*.

Kernel type	Min.	Q_0.25_.	Median	Mean	Q_0.75_.	Max.
Spatially invariant	0.550	0.652	0.705	0.683	0.719	0.735

**Fig 5:**
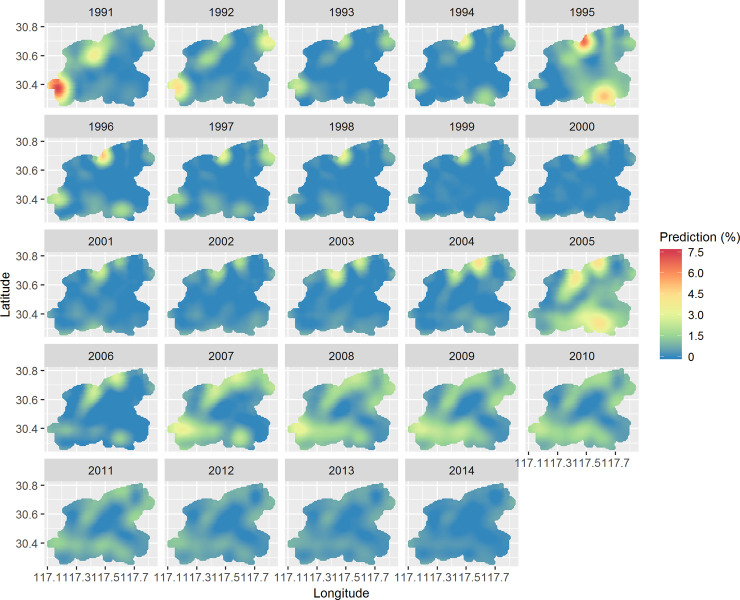
Annual predicted prevalence of schistosomiasis in Guichi, Anhui Province, China, from 1991 to 2014. Predicted prevalence (calculated by percentage) for 1991–2005 were produced using the spatially varying kernel in the IDE spatio-temporal model, while those of 2006–2014 using the spatially invariant kernel. This figure was produced in R 3.4.1 (R Core Team, Vienna, Austria) using shapefiles representing Guichi’s current administrative units (obtained from the geographic data at https://www.naturalearthdata.com/).

**Fig 6:**
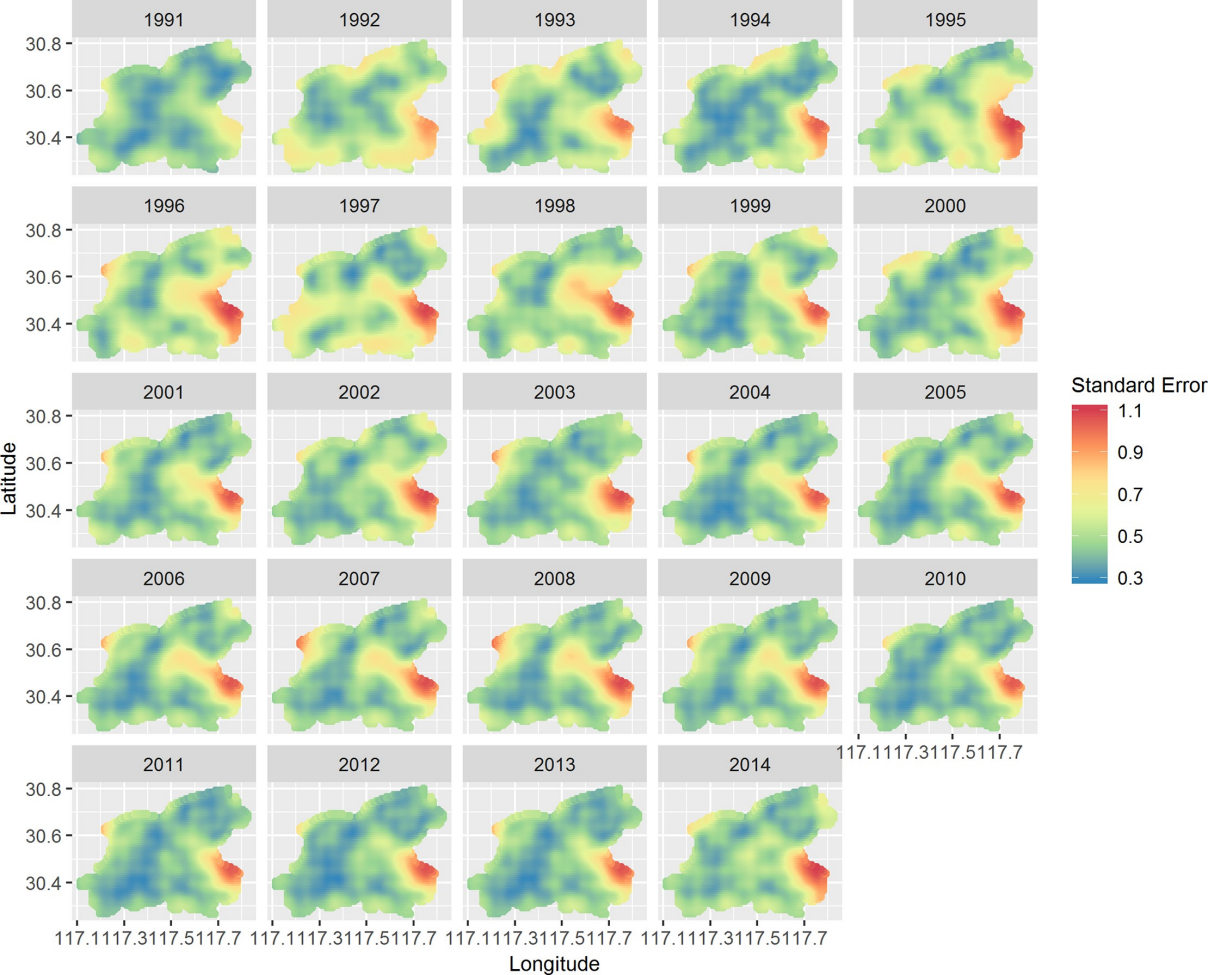
Annual standard error of predicted prevalence of schistosomiasis in Guichi, Anhui Province, China, from 1991 to 2014. Standard errors for 1991–2005 were produced using the spatially varying kernel in the IDE spatio-temporal model, while those of 2006–2014 using the spatially invariant kernel. This figure was produced in R 3.4.1 (R Core Team, Vienna, Austria) using shapefiles representing Guichi’s current administrative units (obtained from the geographic data at https://www.naturalearthdata.com/).

Fig 5 displays the annual map of predicted prevalence for schistosomiasis during the study period. Relatively high prevalence (>4%, shown as strong yellow to red shades) occasionally occurred before 2006. The prevalence, indicated by large patches of light yellow shades (2007–2011) and almost all patches of blue shades (2012–2014), shows a homogenous and decreasing trend over the study area. Fig 6 represents corresponding estimates of the standard error of the predictions. The maps present similar patterns across the study period: a lower level of uncertainty is apparent in locations close to sampled villages while a higher level of uncertainty is present in locations distant from sampled villages. Of note, the standard errors are much lower and more homogenous during 2006–2014 than those during 1991–2005.

## Discussion

A dynamic spatio-temporal model was used to investigate the evolution of the schistosomiasis pattern in a high endemic area of schistosomiasis in China. Compared to the descriptive approach, used in most previous studies [23,32,33,34,35,36] which fits means and covariances to spatio-temporal data, we believe that our model represents a more dynamic approach that accounts for prior knowledge (i.e., shifting trend of prevalence shown in Fig 3) to capture the evolution of current and future spatial fields from the characteristics of past ones. A description of this evolution of schistosomiasis prevalence should be helpful for the understanding of the modalities of the spread of the disease over space and time and can provide useful information for future schistosomiasis monitoring and control.

Hovmoller plots (Fig 3) for both the latitude and longitude coordinates for the schistosomiasis prevalence data provide empirical recognition of the formation of disease distribution patterns as well as the prior knowledge (i.e. propagation of prevalence) for our dynamic modeling. In our case, the prevalence during 1991–2005 shows a fluctuating pattern with higher prevalence present at higher latitudes (located further North), which is possibly due to the Yangtze and Qiupu Rivers in the northern part of the study area (Fig 1), and the potential temporal trends in prevalence with decreasing longitude, probably suggesting propagation through time from the eastern longitudes towards the western longitudes. During 2006–2014, the prevalence show a fairly constant pattern with foci at western longitudes which is probably related to the two rivers (Fig 1) which indicates that there is no shift of schistosomiasis prevalence during this period.

The empirical recognition of the disease pattern formation is supported by results of our dynamic modelling. We used a spatially invariant model to fit spatio-temporal prevalence data after carrying out the model validation. We find that there is nearly no shift present in the disease pattern after the effect of proximity to river is taken into account, suggesting that the effect of proximity to river can capture the spatio-temporal variation of schistosomiasis prevalence data and that water contact is possibly still an important determinant for schistosomiasis as a result of agricultural activities and fishing [20]. The stationary pattern means the disease's pattern remain unchanged/stable over time and often exhibit intermittent areas of high density of cases that favor persistence of the disease. This pattern formation and the accompanying transition is more evident in the annual prevalence predictions (Fig 5). During 1991–2005, schistosomiasis prevalence showed heterogeneities, i.e., limited number of geographic foci of high prevalence (except 2005) occurred intermittently over the study area, whereas homogeneities were present during 2006–2014 as large patches of relatively low prevalence constantly occurred.

Although no evidence of regularly global propagation of the disease was seen, local pattern transition existed which can be potentially explained by the change in schistosomiasis control strategies during the study period [9]. In the early 1990s, the local government of Guichi implemented the 10-year WBLP according to the national strategy for schistosomiasis control, which were largely based on chemotherapy [37]. After conclusion of the WBLP in 2001, the local government continued to carry out this strategy [20] but the disease rebounded afterwards due to limited financial support. This rebound can be seen from maps in 2003–2005 (Fig 5). Since 2006, a revised strategy was implemented according to the new national control strategy [38], which aimed at reducing the role of bovines and humans as infection sources. Specific interventions were as follows: (1) all bovines were removed from the study area (e.g., some farm bovines were replaced with tractors, or transferred to non-endemic regions; some bovines were killed); (2) improved sanitation facilities (e.g., sanitary lavatories and piped water were installed); (3) a health education program was initiated, focusing on avoiding snail-infected areas and associated river water; (4) modifying snail microhabitat through agricultural practices (e.g., modifying crop structures in snail-inhabited areas to reduce water use). This integrated strategy substantially changed the local physical environment as well as human behavior. We, therefore, believe this change of control strategy resulted in the local pattern transition of the disease presented here. Although the integrated strategy is more effective than chemotherapy alone in reducing prevalence (as shown in Fig 5), our analysis (Table 1) indicates that it is more likely for the disease to persist under this strategy.

Two limitations need discussion. One is that our dynamic model only considered the effect of proximity to river (in formula (1)). We should have used some other factors associated with the disease such as climatic data (e.g., precipitation and temperature) and socio-economic data (e.g., GDP and medical resources). We assumed the climatic factors geographically changed little over the study area (i.e., only at a county level) and the climatic conditions during the study period have not changed substantially [39]. In addition, socio-economic data so far are not available at the village-level. Furthermore, the IDE used in our study models the "residual" after discounting what the covariates explain. If the covariates explain the spatio-temporal variation within the response variable, no trends are left in the “residual” which is the case in our study. Nevertheless, inputting those factors into our dynamic model would be warranted in a future study. The other limitation is that the specificity of serological assays and the sensitivity of stool examination tests are not perfect due to the generally low level of infection, and this could result in an imperfect measure of prevalence data of schistosomiasis. Dynamic modeling with diagnostic errors is also our further study direction.

In summary, we built a hierarchical dynamic spatio-temporal model to investigate the evolution of schistosomiasis pattern in an area known for remaining high transmission. Based on results of pattern analysis, we conclude that local pattern transition was probably due to the change in the two national schistosomiasis control strategies and the integrated strategy is more effective than the chemotherapy-based one, with a caution that the disease would be a long-term endemic. Methodologically, the merit of our study is that it provides a good example for modelling high-dimensional spatio-temporal data in an epidemiological study by using kernel functions to reduce data dimension. As more local and national surveillance systems are established, there would be more large-scale datasets available in public health. Undoubtedly, our study augments the methodology for dealing with such problems.

## Supporting information

S1 Text Supporting text containing details of the kernel function. (DOCX)

## References

[1] Utzinger J, Keiser J. Schistosomiasis and soil-transmitted helminthiasis: common drugs for treatment and control. Expert Opin Pharmacother. 2004;5:263–285. 10.1517/14656566.5.2.263 14996624

[2] Bergquist R, Tanner M. Controlling schistosomiasis in Southeast Asia: a tale of two countries. Adv Parasitol. 2010;72:109–144. 10.1016/S0065-308X(10)72005-4 20624530

[3] Chen M, Feng Z. Schistosomiasis control in China. Parasitol Int. 1999;48:11–19. 10.1016/s1383-5769(99)00004-5 11269321

[4] Wang L, Chen H, Guo J, Zeng X, Hong X. A strategy to control transmission of Schistosoma japonicum in China. N Engl J Med. 2009;360:121–128. 10.1056/NEJMoa0800135 19129526

[5] Zhu H, Yap P, Utzinger J, Jia TW, Li SZ, et al Policy Support and Resources Mobilization for the National Schistosomiasis Control Programme in The People's Republic of China. Adv Parasitol. 2016;92:341–383. 10.1016/bs.apar.2016.03.002 27137452 PMC7103126

[6] Zhou XN, Guo JG, Wu XH, Jiang QW, Zheng J, et al Epidemiology of schistosomiasis in the People's Republic of China, 2004. Emerg Infect Dis. 2007;13:1470–1476. 10.3201/eid1310.061423 18257989 PMC2851518

[7] Wang J, Li T, Huang S, Cong W, Zhu X. Major parasitic diseases of poverty in mainland China: perspectives for better control. Infect Dis Poverty. 2016;5:67 10.1186/s40249-016-0159-0 27476746 PMC4967992

[8] Chen XY, Wang LY, Cai JM, Zhou XN, Zheng J, et al Schistosomiasis control in China: the impact of a 10-year World Bank Loan Project (1992–2001). Bull World Health Organ. 2005;83:43–48. doi: /S0042-96862005000100013 15682248 PMC2623468

[9] Hu Y, Xiong C, Zhang Z, Luo C, Ward M, et al Dynamics of spatial clustering of schistosomiasis in the Yangtze River Valley at the end of and following the World Bank Loan Project. Parasitol Int. 2014;63:500–505. 10.1016/j.parint.2014.01.009 24530858

[10] Wu XH, Zhang SQ, Xu XJ, Huang YX, Steinmann P, et al Effect of floods on the transmission of schistosomiasis in the Yangtze River valley, People's Republic of China. Parasitol Int. 2008;57:271–276. 10.1016/j.parint.2008.04.004 18499513

[11] Zhou YB, Liang S, Jiang QW. Factors impacting on progress towards elimination of transmission of schistosomiasis japonica in China. Parasit Vectors. 2012;5:275 10.1186/1756-3305-5-275 23206326 PMC3519747

[12] Yang GJ, Utzinger J, Zhou XN. Interplay between environment, agriculture and infectious diseases of poverty: Case studies in China. Acta Trop. 2015;141:399–406. 10.1016/j.actatropica.2013.07.009 23906612 PMC7117482

[13] Wang LD, Guo JG, Wu XH, Chen HG, Wang TP, et al China's new strategy to block Schistosoma japonicum transmission: experiences and impact beyond schistosomiasis. Tropical Med Int Health. 2009;14:1475–1483. 10.1111/j.1365-3156.2009.02403.x 19793080

[14] Seto EY, Remais JV, Carlton EJ, Wang S, Liang S, et al Toward sustainable and comprehensive control of schistosomiasis in China: lessons from Sichuan. PLoS Negl Trop Dis. 2011;5:e1372 10.1371/journal.pntd.0001372 22039563 PMC3201916

[15] Liu R, Dong HF, Jiang MS. The new national integrated strategy emphasizing infection sources control for schistosomiasis control in China has made remarkable achievements. Parasitol Res. 2013;112:1483–1491. 10.1007/s00436-013-3295-5 23354940

[16] Zhou YB, Liang S, Chen GX, Rea C, He ZG, et al An Integrated Strategy for Transmission Control of Schistosoma japonicum in a Marshland Area of China: Findings from a Five-Year Longitudinal Survey and Mathematical Modeling. Am J Trop Med Hyg. 2011;85:83–88. 10.4269/ajtmh.2011.10-0574 21734130 PMC3122349

[17] Meurs L, Mbow M, Boon N, van den Broeck F, Vereecken K, et al Micro-Geographical Heterogeneity in Schistosoma mansoni and S haematobium Infection and Morbidity in a Co-Endemic Community in Northern Senegal PLoS Neglect Trop Dis. 2013:7. 10.1371/journal.pntd.0002608 PMC3873272 24386499

[18] Woolhouse M, Dye C, Etard JF, Smith T, Charlwood JD, et al Heterogeneities in the transmission of infectious agents: Implications for the design of control programs. Proc Natl Acad Sci U S A. 1997;94:338–342. 10.1073/pnas.94.1.338 8990210 PMC19338

[19] Hu Y, Zhang Z, Chen Y, Wang Z, Gao J, et al Spatial pattern of schistosomiasis in Xingzi, Jiangxi Province, China: the effects of environmental factors. Parasit Vectors. 2013;6:214 10.1186/1756-3305-6-214 23880253 PMC3726341

[20] Zhang Z, Carpenter TE, Chen Y, Clark AB, Lynn HS, et al Identifying high-risk regions for schistosomiasis in Guichi, China: a spatial analysis. Acta Trop. 2008;107:217–223. 10.1016/j.actatropica.2008.04.027 18722565

[21] Hu Y, Xiong C, Zhang Z, Luo C, Cohen T, et al Changing patterns of spatial clustering of schistosomiasis in Southwest China between 1999–2001 and 2007–2008: assessing progress toward eradication after the World Bank Loan Project. Int J Environ Res Public Health. 2014;11:701–712. 10.3390/ijerph110100701 24394217 PMC3924469

[22] Hu Y, Li R, Chen Y, Gao F, Wang Q, et al Shifts in the spatiotemporal dynamics of schistosomiasis: a case study in Anhui Province, China. PLoS Negl Trop Dis. 2015;9:e3715 10.1371/journal.pntd.0003715 25881189 PMC4400088

[23] Hu Y, Li R, Bergquist R, Lynn H, Gao F, et al Spatio-temporal Transmission and Environmental Determinants of Schistosomiasis Japonica in Anhui Province, China. PLoS Negl Trop Dis. 2015;9:e3470 10.1371/journal.pntd.0003470 25659112 PMC4319937

[24] Sun G, Jusup M, Jin Z, Wang Y, Wang Z. Pattern transitions in spatial epidemics: Mechanisms and emergent properties. Phys Life Rev. 2016;19:43–73. 10.1016/j.plrev.2016.08.002 27567502 PMC7105263

[25] Cohen M. Changing patterns of infectious diseases. Nature. 2000;406:762–767. 10.1038/35021206 10963605

[26] Lloyd-Smith JO, Schreiber SJ, Kopp PE, Getz WM. Superspreading and the effect of individual variation on disease emergence. Nature. 2005;438:355–359. 10.1038/nature04153 16292310 PMC7094981

[27] Zhao GM, Zhao Q, Jiang QW, Chen XY, Wang LY, et al Surveillance for schistosomiasis japonica in China from 2000 to 2003. Acta Trop. 2005;96:288–295. 10.1016/j.actatropica.2005.07.023 16202597

[28] Yu JM, de Vlas SJ, Jiang QW, Gryseels B. Comparison of the Kato-Katz technique, hatching test and indirect hemagglutination assay (IHA) for the diagnosis of Schistosoma japonicum infection in China. Parasitol Int. 2007;56:45–49. 10.1016/j.parint.2006.11.002 17188018

[29] Hovmoller E The trough-and-ridge diagram. Tellus 1: 62–66.

[30] Dempster AP, Laird NM, Rubin DB. Maximum Likelihood from Incomplete Data via the EM Algorithm. J R Stat Soc B Meth. 1977;39:1–38.

[31] Harvey AC Forecasting, Structural Time Series Models and the Kalman Filter. Cambridge: Cambridge University Press 1989.

[32] Hu Y, Gao J, Chi M, Luo C, Lynn H, et al Spatio-Temporal Patterns of Schistosomiasis Japonica in Lake and Marshland Areas in China: The Effect of Snail Habitats. Am J Trop Med Hyg. 2014;91:547–554. 10.4269/ajtmh.14-0251 24980498 PMC4155558

[33] Hu Y, Ward MP, Xia C, Li R, Sun L, et al Monitoring schistosomiasis risk in East China over space and time using a Bayesian hierarchical modeling approach. Sci Rep. 2016;6:24173 10.1038/srep24173 27053447 PMC4823756

[34] Karagiannis-Voules DA, Biedermann P, Ekpo UF, Garba A, Langer E, et al Spatial and temporal distribution of soil-transmitted helminth infection in sub-Saharan Africa: a systematic review and geostatistical meta-analysis. Lancet Infect Dis. 2015;15:74–84. 10.1016/S1473-3099(14)71004-7 25486852

[35] Gao F, Ward MP, Wang Y, Zhang Z, Hu Y. Implications from assessing environmental effects on spatio-temporal pattern of schistosomiasis in the Yangtze Basin. China Geospat Health. 2018;13 10.4081/gh.2018.730 30451479

[36] Hu Y, Li S, Xia C, Chen Y, Lynn H, et al Assessment of the national schistosomiasis control program in a typical region along the Yangtze River, China. Int J Parasitol. 2017;47:21–29. 10.1016/j.ijpara.2016.09.003 27866904

[37] Yuan H, Jiagang G, Bergquist R, Tanner M, Xianyi C. The 1992–1999 World Bank schistosomiasis research initiative in China: outcome and prospectives. Parasitol Int. 2005;49:195–207. 10.1016/s1383-5769(00)00045-3 11426575

[38] Li SZ, Luz A, Wang XH, Xu LL, Wang Q, et al Schistosomiasis in China: acute infections during 2005–2008. Chin Med J. 2009;122:1009–1014. 19493433

[39] Guemas V, Doblas-Reyes F, Andreu-Burillo I, Asif M. Retrospective prediction of the global warming slowdown in the past decade. Nat Clim Chang. 2013;3:649–653.

