# Supplementary material for: Dynamic evolution of schistosomiasis distribution under different control strategies: Results from surveillance covering 1991–2014 in Guichi, China
Source: PLoS Negl Trop Dis. 2021 Jan 6;15(1):e0008976. doi: 10.1371/journal.pntd.0008976 (PMC7787434; doi:10.1371/journal.pntd.0008976)
Supplement: S1 Text — (DOCX) [file pntd.0008976.s001.docx]

**Appendix Text 1. Additional information regarding the kernel function**

**Transmission kernel**

As shown in the manuscript, we used a Gaussian-shape transition kernel in the process model as follows:

$m\left( s,x;\theta_{p} \right)=\theta_{p,1}(s)exp(-\frac{1}{\theta_{p,2}\left( s \right)}[\left( x_{1}-\theta_{p,3}\left( s \right)-s_{1} \right)^{2}+\left( x_{2}-\theta_{p,4}\left( s \right)-s_{2} \right)^{2}])$ (S1)

where all the kernel parameters $\theta_{p}$ in this formula are spatially varying or spatially invariate. In order to reduce the dimension of $\theta_{p}$, we use spatial basis functions to decompose the $\theta_{p}$. Specifically, we suppose that $\theta_{p,1}$ and $\theta_{p,2}$ are spatially invariant (usually a reasonable assumption [1]), and use the following bisquare basis functions to decompose $\theta_{p,3}$ and $\theta_{p,4}$:

${b(s,v)\equiv\{}_{0, otherwise}^{{\{1-{\{\left\| v-s \right\|/r\}}^{2}\}}^{2}, \left\| v-s \right\|\leq r}$ (S2)

where $r$ is the aperture parameter and $s$ and $v$ are locations. In our study, we constructed 12 spatial basis functions with one resolution, using FRK package of R [2]. Another way to reduce the dimension of $\theta_{p}$ is to suppose $\theta_{p,3}$ and $\theta_{p,4}$ are spatially invariant as well, which is a complete spatially invariant kernel as follows:

$m\left( s,x;\theta_{p} \right)=\theta_{p,1}exp(-\frac{1}{\theta_{p,2}}[\left( x_{1}-\theta_{p,3}-s_{1} \right)^{2}+\left( x_{2}-\theta_{p,4}-s_{2} \right)^{2}])$ (S3)

In summary, the number of parameters in formula (S1) and (S3) are 26 (i.e., $\theta_{p,1}$, $\theta_{p,2}$, 12 $\theta_{p,3}$, and 12 $\theta_{p,4}$) and 4 (i.e., $\theta_{p,1}$, $\theta_{p,2}$,$\theta_{p,3}$, and $\theta_{p,4}$), respectively.

**References**

1. Wikle CK, Zammit-Mangion A, Cressie N: **Dynamic Spatio-Temporal Models**. In: *Spatio-Temporal Statistics with R.* New York: Chapman and Hall/CRC; 2019.

2. Zammit-Mangion A, Cressie N: **Fixed Rank Kriging: The R package**. In*.*; 2018.
